# Quality Aspects of Designing Prohealth Liver Sausages Enriched with Walnut Paste

**DOI:** 10.3390/foods11243946

**Published:** 2022-12-07

**Authors:** Tomasz Florowski, Anna Florowska, Marta Chmiel, Lech Adamczak, Dorota Pietrzak, Agnieszka Ostrowska, Iwona Szymańska

**Affiliations:** 1 Department of Food Technology and Assessment, Institute of Food Sciences, Warsaw University of Life Sciences-SGGW, 159c Nowoursynowska Street, 02-787 Warsaw, Poland; 2 Department of Nanobiotechnology, Institute of Biology, Warsaw University of Life Sciences-SGGW, 8 Ciszewskiego Street, 02-786 Warsaw, Poland

**Keywords:** liver sausages, walnuts, enriching, physicochemical characteristics

## Abstract

The aim of the study was to determine the influence of enriching liver sausages with different levels of walnut paste on the quality properties of this product. Sausages were produced with 5, 10, 15, 20, and 25% amount additions of walnut paste and without the addition of nuts (control product). It was found that walnut paste, especially when introduced at an amount >15%, was a component that limited thermal losses and significantly modified the characteristics of liver sausages. The addition of walnut paste also increased the fat content of liver sausages by two–three times, which was one of the factors that weakened their structure, including lowering their compression, shear, and penetration force but increasing their spreadability. Moreover, the addition of walnut paste at an amount of ≥20% resulted in the products having a slightly different color, with lower values for the a* color parameter. Such changes were assessed as a favorable modification to the product, increasing its overall desirability, especially with the addition of walnut paste at the level of 20%. Walnut paste can therefore be a valuable ingredient that allows for the development of a health-promoting product with improved quality features. However, with the addition of a walnut paste at an amount of 25%, it is necessary to take into account the more rapid and unfavorable fat changes that occur during the storage of the liver sausages, as indicated by about 50% higher TBARS values (compared to the control product).

## 1. Introduction

Liver sausage is a product popular in many countries, made from precooked meat, fat, liver, spices, and optionally a variety of flavors and technological additives. It is produced in wide varieties, differing in the type of raw materials used. Many types of liver sausages have a traditional, regional character [1,2,3]. A common characteristic feature of liver sausages is a soft and spreadable structure, resulting from a relatively high (25–40%) fat content [1,2,4,5]. Unfortunately, this high-fat content in liver sausages, apart from having a positive effect on the structure and sensory properties (e.g., flavor), means that such products may be negatively perceived by consumers. Fat plays a decisive role in product properties and consumer acceptance [6,7]. Meat and meat products, especially those with a high-fat content and that are rich in SFA (saturated fatty acids), are often perceived as dietary components that may contribute to the occurrence of cardiovascular diseases or the development of certain types of cancer [8,9,10,11]. Such an image of meat products among consumers prompts producers to look for methods to improve their health value and to design innovative, prohealthy meat products [9,10,12,13,14]. Despite the fact that it would be more expensive than a traditional product, such a product could be appreciated by buyers looking for meat products from the functional food segment. In the case of such products, their price may not be a decisive factor in making purchasing decisions [15]. For such products, taste, product quality, convenience, and the credibility of health claims are important factors [16]. An interesting concept to increase the health value of processed meat may be including walnuts in their raw material composition [17,18]. Walnuts are characterized by a high-fat content (62–68%), with a nutritionally beneficial composition of fatty acids. The walnut fat contains a high (approx. 12–15% of total fatty acids) content of alpha linolenic acid [19,20,21,22]. Its consumption has an important role in the prevention and treatment of, among others, coronary artery disease, hypertension, diabetes, arthritis, and cancer [23,24,25]. In addition, the high prohealth value of walnuts is associated with their favorable amino acid composition (protein rich in arginine), fiber content, antioxidant vitamins, and microelements [21,26,27,28]. The high prohealth value of walnuts has become the basis for authorizing health claims on products enriched with them on the UE market. The effect indicated within the health claims is obtained with the consumption of nuts at the level of 30 g/day [29].

Research on the possibility of using walnuts to increase the health value of processed meat has so far been carried out on restructured beef steak [18,30], frankfurters [31], and dry fermented sausage [27]. The incorporation of walnuts into meat products allows for products with a favorably modified composition to be obtained when compared to traditional products, including elevated levels of PUFA (polyunsaturated fatty acids) and reduced ratio of n-6 to n-3 fatty acids [18,27,30,31]. The addition of walnuts also causes some changes in the proportion of amino acids. Significant changes were observed, especially in the content of valine, alanine, arginine, which increased after the addition of walnuts, and tyrosine, the level of which decreased under these conditions. The lysine/arginine ratio also decreased, which is a positive change that contributes to the reduction in atherosclerosis [30]. Moreover, it also allows for obtaining a higher content of tocopherol and a nutritionally favorable profile of lysine/arginine in the product. The authors suggested, therefore, that such products could be used as functional meat products [30,31].

When designing prohealth meat products with the addition of walnuts, it should be taken into account that this ingredient may have a significant impact on the characteristics of the created products, especially their structure. According to the research of Jiménez-Colmenero et al. [19], Cofrades et al. [32], Serrano et al. [33], and Serrano et al. [34], the addition of walnuts to the batter of restructured beef steaks caused, among other factors, a significant reduction in shear force and bind strength. Products that received an addition of nut content were characterized by softer and less cohesive structures. As the authors indicated, such an impact on the texture could be the result of the deposition of nut material particles on the meat pieces, which made it difficult to combine them while creating the batter. It also made the gelling process more difficult, and the created protein network structure became less continuous and more dispersed. Ayo et al. [35] state that in the case of frankfurters, this effect was different. Products with the addition of 25% of walnuts obtained the highest values of hardness and chewiness and the lowest values of springiness in comparison to a traditional product. This indicates that the influence of walnuts on the quality of products may depend on the characteristics of the meat–fat system, the amount of added nuts, the technological additives used to facilitate the creation of a stable structure of the meat product (gel network), and the technology of production. So far, the influence of the addition of walnuts on the physicochemical and sensory properties of meat products that are made from heat-treated meat (such products include, among others, liver sausage) has not been analyzed. In the case of a liver sausage type product, the mechanism of obtaining appropriate product characteristics, especially the structure of the product, is different than in the case of restructured products and frankfurters, which results from the denaturation of muscle proteins before the batter formation stage [1,2,36]. Therefore, the aim of the study was to determine the influence of enriching liver sausage with different levels of walnut paste on the quality properties of this product.

## 2. Materials and Methods

### 2.1. Materials and Production of Liver Sausages

The raw meat for the production of the liver sausages was beef shoulder and flank. The meat purchased once from a local producer was cut into strips, vacuum packed, frozen to −20 °C, and stored until usage (approximately 4 weeks). Before processing, the meat was thawed (24 h, 4–6 °C). Next, the meat was cooked in water (85–90 °C, 90 min) and ground in a meat grinder (mesh with ø 4.5 mm holes; Mesko AL. 2-4, Mesko-AGD Sp. z o.o., Skarżysko-Kamienna, Poland). The meat broth was obtained when the cooked beef shoulder was used as a recipe ingredient for the batter. Beef liver, which was also used for the production of the liver sausages, was ground in a grinder (mesh with ø 4.5 mm holes) and homogenized in a heavy-duty bowl cutter with rapidly moving blades of 3000 rpm (Stephan Machinery GmbH, Hameln, Germany), with the addition of 2% sodium chloride for 30 s. Eggs, purchased in local stores, were cracked immediately before production and mixed in a mixer (Kenwood Major type KM 800, Kenwood Ltd., Birmingham, England) until a uniform emulsion was obtained.

The basic batter for the production of the liver sausages consisted of 58% of cooked beef (shoulder and flank meat mixed in a proportion 1:1), 6% of homogenized beef liver, 4% egg mass, and 32% meat broth. Ingredients, such as sodium chloride (1.0%) and pepper (0.085%; Kamis, Poland), were also added; their amount was calculated in relation to the weight of the batter. Six products were manufactured: a control (WAL/0), which did not contain walnut paste, and five that had walnut paste added. The walnut paste was added in the amount calculated in relation to the weight of the basic batter, that is, in the amount of 5 (WAL/5), 10 (WAL/10), 15 (WAL/15), 20 (WAL/20) and 25% (WAL/25). The amount of walnut paste added to the batter was determined on the basis of the results of previous studies on the effect of the addition of walnuts on the quality of other meat products [19,32,35,37] and on the basis of preliminary studies. The nut paste was prepared directly before the batter production by grinding the nuts (Bakalland S.A., Warszawa, Poland) in a laboratory mill (WŻ-1, Zakład Badawczy Przemysłu Piekarskiego Sp. z o.o., Bydgoszcz, Poland) to a particle size of <25 µm.

**Figure 1:**
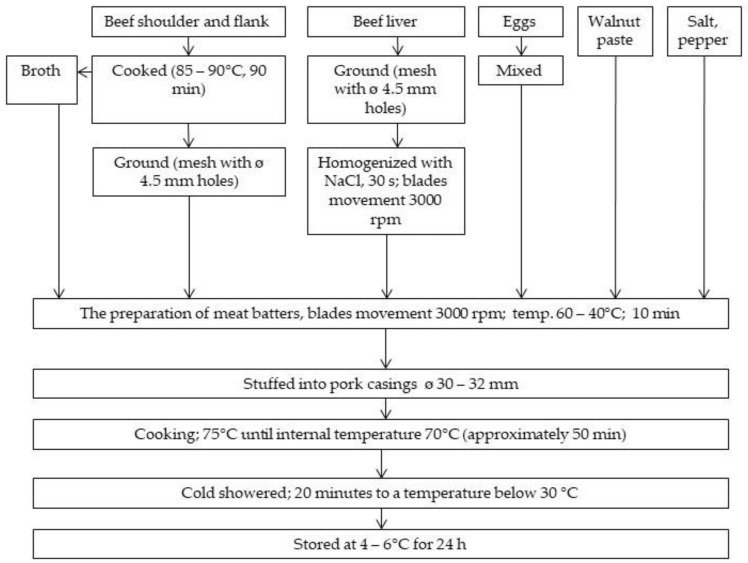
Technological scheme for the production of liver sausage with the addition of walnut paste.

The preparation of the meat batters consisted of two stages, which were carried out in a heavy-duty bowl cutter with rapidly moving blades of 3000 rpm (Stephan Machinery GmbH, Hameln, Germany) (Figure 1). In the first stage, the cooked meat (temperature approximately 60 °C) was homogenized for 5 min with added broth (temperature approximately 60 °C), additives, and, optionally, walnuts (exception WAL/0 sample). In the next stage, the homogenized beef liver and egg mass was added and homogenized again for 5 min. The end temperature of the homogenized batter was approximately 40 °C. After that time, the batters were stuffed into pork casings (Kadek sp. z o.o., Żukowo, Poland) of a diameter of 30–32 mm, using a hand sausage stuffer (Friedr, Dick GmbH & Co. KG, Deizisau, Germany) and batters of about 150 mm (in length) were formed. Then, the batters were heated in a scalding chamber (Jugema, Środa Wielkopolska, Poland) at 75 °C until their internal temperature reached 70 °C (time approximately 50 min). Subsequently, the products were cold showered for 20 min to a temperature below 30 °C. After cooling, the liver sausages were stored at 4–6 °C for 24 h. After that time, a quality evaluation of the products was performed. Four replications of the experiment were conducted at separate times.

### 2.2. Methods

#### 2.2.1. Determination of Cooking Loss

The amount of mass loss generated during the thermal treatment was determined on the basis of the difference in the liver sausage mass before the thermal treatment process and after cooling. The result was expressed as a percentage of the weight of the liver sausage before heat treatment.

#### 2.2.2. Determination of Drip Loss after Cooling Storage

Determination of the drip loss after cooling storage was determined according to Florowski et al. [18], with time modification (taking into account the characteristics of the product). The samples of liver sausage (one baton weighing approximately 100 g) were vacuum packed (Multivac A 200/15, Multivac Sp. z o.o., Natailn, Poland) in polyethylene bags and stored under cooling conditions (4–6 °C) for 14 days. After, the storage samples were removed from their packages and blotted dry with filter paper. Drip loss was expressed as a percent of initial sample weight.

#### 2.2.3. Measurement of Water Activity

A sample of the product was placed in the measuring vial and then in the apparatus: Aqua Lab (CX-2, Decagon Devices Inc., Pullman, WA, USA). The measurement was carried out at 25 ± 1.5 °C.

#### 2.2.4. Determination of Basic Chemical Composition

The moisture, protein, and fat content of the liver sausages were determined by AOAC Official Method 2007.04 [38], applicable to meat and meat products using the FOSS FoodScan™ (FOSS Analytical, Warsaw, Poland) near-infrared (NIR) spectrophotometer with the FOSS Artificial Neural Network (ANN) calibration model and associated database.

#### 2.2.5. Product Storage Conditions and Determination of TBARS Indicator

The samples of liver sausage (one baton weighing approximately 100 g) were vacuum packed (Multivac A 200/15, Multivac Sp. z o.o., Natailn, Poland) in polyethylene bags and stored under cooling conditions (4–6 °C) for 14 days. After this period, the TBARS indicator was determined using the modified method of Shahidi [39]. Absorbance was measured at 532 nm using a spectrophotometer (Hitachi U-1100; Gemini bv., Apeldoorn, The Netherlands) against a blank containing 5 mL of 0.02 M 2-thiobarbituric acid and 5 mL of 10% trichloroacetic acid. The TBARS indicators were reported as malondialdehyde (MDA) equivalents (mg MDA/kg of product) using a standard curve with malonaldehydebis (dimethylacetal). The conversion factor was 2.34.

#### 2.2.6. Measurement of pH Level

The pH level was measured by using a pH meter (CP-411, Elmetron, Zabrze, Poland) with a glass calomel electrode. Before the measurement, 10 g of the ground final products were blended with 30 mL of distilled water.

#### 2.2.7. Measurement of Product Texture and Rheological Parameters

The texture and rheological parameters of the liver sausages were measured after removing the casings from the sausages at a temperature of 20 °C. The measurement was repeated four times, taking the mean value as the final result. For compression, shear, and penetration force measurement, a Zwicki 1120 (Zwick GmbH & Co., Ulm, Germany) universal testing machine was used. The speed of the movement of the measuring head was 50 mm/min. The samples for the compression force measurement were slices of the product with a height of 20 mm. The maximum force needed to compress the samples by 30% of their original height was measured. The samples for measuring the shear force were the baton of the product. The maximum force needed to cut the sample was measured by using a Warner-Bratzler adapter. For the penetration force measurements, slices of the products with a height of 20 mm were used. The maximum force required for the immersion (inside the samples) of a metal, flat-felled mandrel (of a diameter of 13 mm and a depth of 10 mm) was measured. For the spreadability and adhesiveness measurements, a TA.XT texture analyzer (TA.XT Plus, Stable Micro Systems, Godalming, UK) with a 5 kg load cell was used. The probe used for spreadability measurement was a TTC Spreadability Rig. The test speed and distance were set to 3.0 mm/s and 20 mm, respectively. The values of the spreadability and adhesiveness were analyzed from the graphs using Exponent (version 6.1.4.0) equipment software. The apparent viscosity was measured using Rheotest-2 apparatus (VEB MLW Prüfgeräte-Werk Medingen, Ottendorf-Okrilla, Germany) using a measuring set H/H and a shear rate D = 16.2 s^−1^.

#### 2.2.8. Microstructure

The microstructure of the liver sausages was visualized with a scanning electron microscope—SEM (type FEI, Quanta 200, Jeol, Tokyo, Japan) in the low vacuum mode. The large field detector at an accelerating voltage of 20 kV was used. The images of the microstructure were recorded at 1000× magnification. An analysis of images was performed using a program provided by the FEI Company.

#### 2.2.9. Measurement of L*, a* and b* Color Components

The L*, a*, and b* color components were determined in CIEL*a*b* scale at the surface of the freshly cut liver sausages, using a Minolta CR-200 colorimeter (Konica Minolta, Wrocław, Poland; light source D65, observer 2°, a measuring head hole 8 mm). Each measurement was performed 6 times. The mean value was used as the final result. In order to determine the differences in color between the control product and products with the addition of walnuts, the parameter of the total color difference, ΔE, was also calculated [40] based on the formula presented below.

$$\Delta E=\sqrt{{\left(\Delta L^{*}\right)}^{2}+{\left(\Delta a^{*}\right)}^{2}+{\left(\Delta b^{*}\right)}^{2}}$$

ΔL*, Δa*, Δb*—differences between the color components of WAL/0 and the products with added walnut paste.

#### 2.2.10. Semiconsumer Assessment of Product Desirability

The overall desirability of the products was assessed by a group of 50 people, men and women, aged from 20 to 50 years (it was assumed that the panelists were evenly distributed in terms of age and gender) [41]. The material for assessing the desirability was a bar of the product cut to show the appearance of its cross-section. The panelists were asked to make an overall assessment of the product, taking into account those characteristics that they usually pay attention to when making decisions about the purchase of liver sausages in retail outlets (including the general appearance of the product, its color, structure, and softness). The panelists marked the result of the evaluation on a 10-point scale with a range from 0, which meant an undesirable product that the evaluator would not like to buy, to 10, which meant a very desirable product that the evaluator would very much like to buy.

#### 2.2.11. Statistical Analysis

The results were analyzed using Statistica 13.3 (TIBCO Software Inc., Palo Alto, CA, USA). To determine the significance of the differences between the mean values of the quality characteristics of the liver sausages, a one-way analysis of variance (One-Way ANOVA) was used. Significant differences between treatments were verified using HSD Tukey’s test at a significance level of α = 0.05.

## 3. Results and Discussion

### 3.1. The Effect of Walnuts Addition on Cooking Loss and Drip Loss after the Cool Storage and Water Activity of the Liver Sausages

The incorporation of walnut paste into the liver sausages of an amount of 10% or higher resulted in a significant (*p* < 0.05) reduction in cooking loss during the thermal treatment in relation to the product without walnut addition (Table 1). The higher the level of the addition of walnut paste, the lower the amount of cooking loss from the product. Such a beneficial effect of the addition of walnuts on the amount of cooking loss was also observed by Jiménez Colmenero et al. [19] and Serrano et al. [33] in research on restructured beef steaks. The authors indicated that this effect could be related to the reduction in water content in the batter due to the addition of nuts.

The conducted research also checked how the addition of walnut paste would affect the drip loss after the cool storage of the products. It was found that the addition of walnut paste to the batter of the liver sausages, even for the largest amount used, had no significant effect (*p* > 0.05) on drip loss during refrigerated storage (Table 1). The issue of the effect of the addition of walnuts on the amount of drip loss after the cool storage of the meat products was also analyzed by Serrano et al. [37]. However, the authors showed that, in the case of restructured beef steaks, the addition of walnuts (in the amount of 10 and 20%) resulted in a reduction in the amount of drip loss after cool storage. As the authors pointed out, this effect could be the result of lower water content in the products containing walnuts compared to the control products.

When analyzing the effect of the addition of walnuts on the water activity of the product, it was found that despite the significant differences in the number of weight losses during the thermal treatment between the products with different amounts of walnut addition, the addition of nut paste did not have a significant (*p* > 0.05) effect on this feature in the final product (Table 1).

### 3.2. The Effect of Walnuts Addition on Basic Chemical Composition, the TBARS Indicator, and the pH Level of the Liver Sausages

The addition of walnut paste caused significant changes in the chemical composition of the liver sausages (Table 2). There was a significant (*p* < 0.05) reduction in water content, an increase in fat content (with the addition of nut paste ≥ 10%), and a reduction in the protein content (with 25% addition of nut paste) in the liver sausages, compared to the control product. A similar effect of the addition of walnuts on the content of water, protein, and fat was observed by Serrano et al. [30,34,37] in the research on the effect of the addition of nuts on the quality of restructured beef steak.

Undoubtedly, increasing the fat content of the product (by introducing walnut paste) has a positive effect on the health value of the product, as it increases the content of fat that is rich in PUFA [30,31]. Therefore, such products can be classified as prohealth meat products. However, the unfavorable effect of such modifications may be the increased susceptibility of the products to changes occurring during refrigerated storage, resulting from the high susceptibility of polyunsaturated fatty acids to oxidation. On the basis of the conducted research, it was found that introducing walnut paste up to an amount of 20% into the batter of the liver sausages did not significantly increase the TBARS value during the 14-day refrigerated storage of the product. However, the largest addition (i.e., 25%) resulted in a significant (*p* < 0.05) increase in the TBARS index (Table 2). This indicates that the production of liver sausages with such a large addition of walnut paste may require the simultaneous addition of antioxidants to protect the lipid fraction of the products against negative changes. A similar, unfavorable effect of the addition of walnuts on the value of the TBARS index was found by Serrano et al. [34] in their research on restructured beef steaks.

The research also analyzed the effect of the addition of walnut paste on the pH of the product. It was found that this addition (even with the largest amount of paste) to the batter did not result in significant (*p* > 0.05) changes in the product’s pH (Table 2). The lack of a significant effect with the addition of walnuts on the pH of the product was also indicated by Jiménez Colmenero et al. [19] and Cofrades et al. [32] with restructured beef steaks and Ayo et al. [35] with frankfurter sausages, as well as Ercoskun and Demirci-Ercoskun [27] with fermented sausage. In turn, Serrano et al. [34] and Salejda et al. [42] found that walnut addition causes a slight increase in the pH of the restructured products and cooked steaks, respectively.

### 3.3. The Effect of Walnut Addition on the Texture, Rheological Parameters, and Microstructure of Liver Sausages

**Figure 2:**
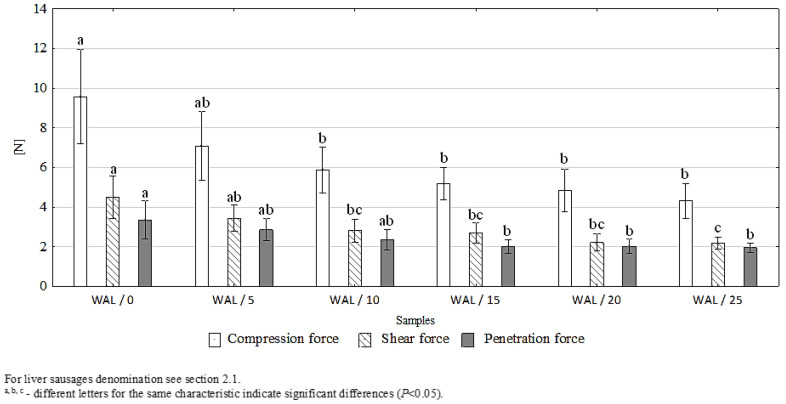
Compression, shear, and penetration force of the liver sausages, *n* = 4.

**Figure 3:**
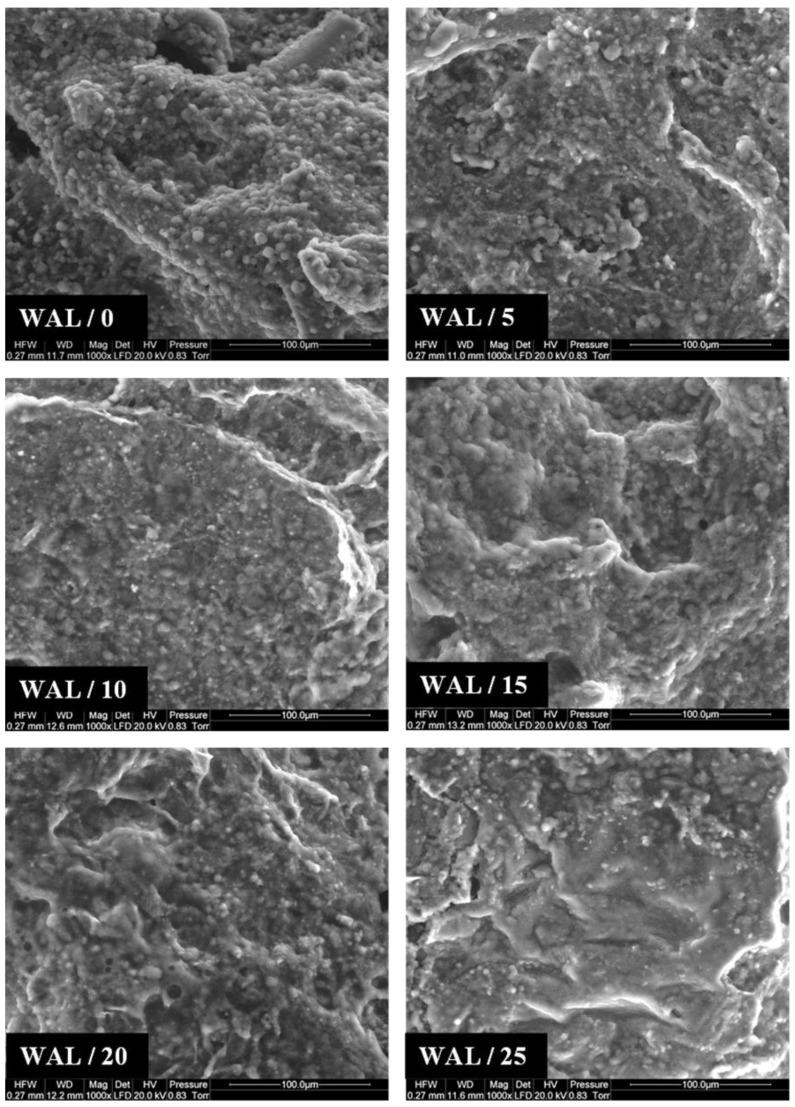
Selected scanning electron micrographs of the liver sausages.

The introduction of walnut paste into the batter significantly weakened the structure of the products. It was found that, when compared to the control product, the compression and shear force of the liver sausages was reduced (with the addition of walnut paste ≥ 10%) and also a reduction in their penetration force (with the addition of walnut paste ≥ 15%) was found (Figure 2). It was also found that the addition of walnut paste (with the addition of ≥20%) to the batter caused a significant (*p* < 0.05) increase in spreadability and a decrease in apparent viscosity (with the addition of ≥10%). However, it did not affect the adhesiveness of the product, even with the highest addition of walnut paste, i.e., 25% (Table 3). Probably, the changes in the rheological characteristics of the product, as a result of introducing walnut paste into the stuffing, were the result of an increase in the fat content of the product (a two–three-fold increase for sausages with walnut paste added, compared to the control product: Table 2). The fat incorporated into the stuffing, together with the nut paste, was found between the particles of the denatured meat, weakening the structure of the product and increasing its spreadability, which was also confirmed by the photos of the microstructure of the products (Figure 3). Increasing the nut content resulted in a product with a smoother and more homogeneous structure.

According to Jiménez Colmenero et al. [19], Cofrades et al. [32], and Serrano et al. [33,34], the weakening of the structure of meat products due to the addition of nuts may result from several factors, including increased fat content, the diluting effect of walnuts in a meat protein system, and possible interferences in meat protein gelation processes. To some extent, this can cause limited binding between meat pieces. At the same time, it should be noted that the weakening of the product structure due to the addition of walnuts may be considered a phenomenon not fully beneficial, as was the case of the restructured beef steaks examined by the cited authors; however, in the case of liver sausages, it should be treated as a positive modification. This type of product should have a delicate and soft structure that makes it easier to spread on bread. In addition, increasing the softness and spreadability of the liver sausages, thanks to the addition of walnuts, may be helpful in the development of innovative meat products for people with special nutritional requirements related to problems with chewing and swallowing products (e.g., the elderly) [43].

### 3.4. The Effect of Walnut Addition on the Color Parameters of the Liver Sausages

The addition of walnut paste to the batter of the liver sausages did not have a significant effect (*p* > 0.05) on the L* and b* color parameters of the products (Table 4). It was found, however, that the addition of ≥20% walnut paste caused a significant (*p* < 0.05) reduction in the value of the a* color parameter of the product when compared to the control product.

When analyzing the total color difference parameter (Table 4), it was found that the addition of up to 10% of walnut paste caused a small change in the color of the liver sausage, noticeable only to the experienced observer (1 < Δ*E* < 2). With higher additions of walnut paste to the batter, the average values of the ΔE parameter (determined between sausage color with the addition of walnut paste and the control product) were in the range of 2 < Δ*E* < 3.5, which indicates that the differences in color between these products were also visible to unexperienced observers [40]. When comparing the obtained results with the results of other authors’ studies, it can be concluded that, in the case of other types of meat products, the addition of walnuts had a greater impact on the color than in the case of liver sausages. For example, Jiménez Colmenero et al. [19] and Cofrades et al. [32] found that the addition of walnuts lowered the L* color parameter and increased the a* and b* color parameters in restructured beef steaks. Ayo et al. [35] found that, for frankfurters, the addition of walnuts was characterized by a higher value for the a* and b* color parameters when compared to traditional products, and also reduced fat content was observed.

### 3.5. The Effect of Walnuts Addition on the Semiconsumer Assessment of Product Desirability

**Figure 4:**
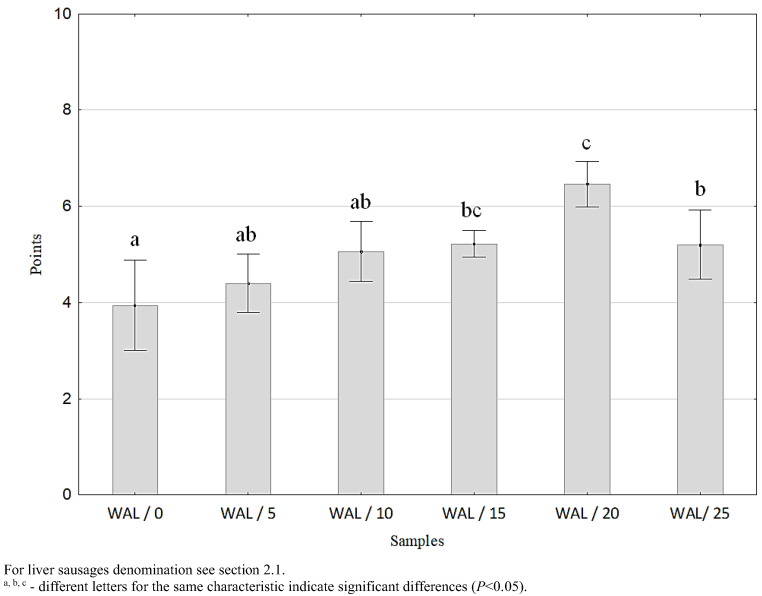
Overall desirability of liver sausages, *n* = 4.

In order to determine how the addition of walnut paste will affect the perception of liver sausage consumers, a semiconsumer assessment of its desirability as a commercial product was carried out. During the evaluation, the panelists focused on those characteristics of the liver sausage, which they usually analyze when making a purchase decision for such a product (the result consisted of one score as an indicator of the overall desirability of the product). On the basis of the obtained results, it was found that the addition of walnut paste to the batter, especially in the amount of ≥15%, resulted in a significant (*p* < 0.05) increase in the ratings awarded during the assessment of the desirability of the product (Figure 4). This could have been the result of various factors, including consumers’ awareness that the assessed product had been enriched with an ingredient with a proven beneficial effect on health [44] and that it was characterized by more favorable (in their opinion) features compared to a traditional product, e.g., a different color, greater softness, etc.

The issue of the influence of the addition of walnuts on the overall desirability of meat products was investigated by, among others, Serrano et al. [37]. The authors showed that the addition of nuts had a positive effect on the overall desirability of restructured beef steaks. In contrast, Jiménez-Colmenero, et al. [19] and Ayo et al. [35] found no significant differences in the overall acceptability of walnut products and traditional products. This indicates that the effect of the addition of walnuts on the desirability of meat products may depend on the type of meat product.

## 4. Conclusions

Walnut paste can be used for the design of a prohealth liver sausage meat product with favorable quality features. Walnut paste, as an ingredient with a high-fat content, especially when added to the batter in a greater amount (i.e., >15%), can cause a weakening of the structure, including a reduction in hardness and increased spreadability, and a change in the color (lowering the value of color parameter a*), all of which are favorable for this type of product. It also causes a significant reduction in the amount of cooking loss in the products, which is economically advantageous. On the other hand, the negative aspect of liver sausage production using a large amount of walnut paste (i.e., 25%) is the faster change of the fats during refrigerated storage, as indicated by higher TBARS index values. This effect should be taken into account in the development of this type of product.

## Figures and Tables

**Table 1:** The cooking loss, drip loss after cool storage, and water activity of the liver sausages (mean value ± standard deviation), *n* = 4.

Type of Liver Sausage	Cooking Loss (%)	Drip Loss (%) after Cooling Storage	Water Activity
WAL/0	9.9 ^c^ ± 0.6	1.37 ± 0.26	0.975 ± 0.006
WAL/5	8.8 ^c^ ± 0.5	1.14 ± 0.17	0.976 ± 0.007
WAL/10	7.1 ^b^ ± 0.5	1.05 ± 0.20	0.976 ± 0.005
WAL/15	7.0 ^b^ ± 1.1	1.11 ± 0.21	0.973 ± 0.008
WAL/20	6.0 ^ab^ ± 0.4	1.14 ± 0.30	0.973 ± 0.005
WAL/25	5.4 ^a^ ± 1.1	1.07 ± 0.32	0.973 ± 0.004

For liver sausages denomination, see Section 2.1. ^a–c^—different letters in the same column indicate significant differences (*p <* 0.05).

**Table 2:** Basic chemical composition, TBARS indicator values, and pH level of the liver sausages (mean value ± standard deviation), *n* = 4.

Type of Liver Sausage	Moisture (g/100 g)	Protein (g/100 g)	Fat (g/100 g)	TBARS (mg MDA/kg of Product)	pH
WAL/0	69.6 ^e^ ± 0.9	24.0 ^b^ ± 1.8	5.6 ^a^ ± 1.6	0.91 ^a^ ± 0.18	6.13 ± 0.03
WAL/5	66.9 ^d^ ± 1.7	24.2 ^b^ ± 1.8	8.1 ^ab^ ± 1.3	0.63 ^a^ ± 0.16	6.16 ± 0.03
WAL/10	64.2 ^c^ ±1.3	23.3 ^b^ ± 1.7	10.5 ^bc^ ± 1.3	0.76 ^a^ ± 0.15	6.15 ± 0.02
WAL/15	61.3 ^b^ ±1.1	22.6 ^ab^ ± 1.1	12.9 ^cd^ ± 1.9	0.96 ^a^ ± 0.20	6.14 ± 0.03
WAL/20	59.8 ^ab^ ± 1.1	21.6 ^ab^ ± 0.8	14.4 ^d^ ± 1.9	1.05 ^ab^ ± 0.21	6.11 ± 0.04
WAL/25	57.9 ^a^ ± 1.5	20.4 ^a^ ± 0.5	16.2 ^d^ ± 2.2	1.44 ^b^ ± 0.35	6.13 ± 0.04

For liver sausages denomination, see Section 2.1. ^a–e^—different letters in the same column indicate significant differences (*p <* 0.05).

**Table 3:** Spreadability, adhesiveness, and apparent viscosity of the liver sausages (mean value ± standard deviation), *n* = 4.

Type of Liver Sausage	Spreadability (N)	Adhesiveness (N)	Apparent Viscosity (Pa*s)
WAL/0	48.3 ^b^ ± 1.8	−22.1 ± 3.4	1494 ^b^ ± 174
WAL/5	43.6 ^ab^ ± 3.9	−22.1 ± 1.7	1258 ^ab^ ± 166
WAL/10	42.8 ^ab^ ± 2.8	−21.7 ± 1.0	1145 ^a^ ± 179
WAL/15	40.0 ^ab^ ± 3.3	−21.4 ± 1.9	1121 ^a^ ± 166
WAL/20	38.9 ^a^ ± 2.8	−19.9 ± 1.1	1073 ^a^ ± 115
WAL/25	38.7 ^a^ ± 5.0	−19.3 ± 1.2	998 ^a^ ± 100

For liver sausages denomination, see Section 2.1. ^a,b^—different letters in the same column indicate significant differences (*p* < 0.05).

**Table 4:** Color components and Δ*E* of the liver sausages (mean value ± standard deviation), *n* = 4.

Type of Liver Sausage	L*	a*	b*	Δ*E*
WAL/0	58.42 ± 3.30	9.10 ^b^ ± 0.89	10.86 ± 1.37	-
WAL/5	57.73 ± 2.16	8.83 ^ab^ ± 1.04	10.64 ± 0.79	1.69 ± 0.84
WAL/10	58.59 ± 2.06	8.39 ^ab^ ± 0.83	10.74 ± 1.12	1.93 ± 0.62
WAL/15	59.07 ± 1.07	7.98 ^ab^ ± 0.75	10.99 ± 0.78	2.45 ± 0.70
WAL/20	59.03 ± 1.88	7.71 ^a^ ± 0.84	11.24 ± 0.77	2.70 ± 0.77
WAL/25	59.35 ± 1.35	7.46 ^a^ ± 0.77	11.34 ± 0.69	3.27 ± 0.72

For liver sausages denomination, see Section 2.1. ^a,b^—different letters in the same column indicate significant differences (*p* < 0.05).

## Data Availability

Data is contained within the article.
